# InsectFish—The Use of Insect Meal in the Fish Sector in Creating Farm-to-Fork Value: Chemical and Quality Characteristics of *Sparus aurata* Fillets Fed *Hermetia illucens* Larvae-Based Feed

**DOI:** 10.3390/foods14173107

**Published:** 2025-09-05

**Authors:** Emma Copelotti, Giovanni Sogari, Giulia Andreani, Baldassare Fronte, Roberta Moruzzo, Chiara Sangiacomo, Asia Zanzot, Andrea Serra, Giuliana Parisi, Isabella Tucciarone, Liudmyla Fihurska, Manus Carey, Katrina Campbell, Simone Mancini

**Affiliations:** 1 Department of Veterinary Sciences, University of Pisa, Viale delle Piagge 2, 56125 Pisa, Italy; emma.copelotti@phd.unipi.it (E.C.); baldassare.fronte@unipi.it (B.F.); roberta.moruzzo@unipi.it (R.M.); chiara.sangiacomo@phd.unipi.it (C.S.); asia.zanzot@phd.unipi.it (A.Z.); 2 Department of Food and Drug, University of Parma, Parco Area delle Scienze 45, 43124 Parma, Italy; giovanni.sogari@unipr.it (G.S.); giulia.andreani@unipr.it (G.A.); 3 Interdepartmental Research Center Nutrafood “Nutraceuticals and Food for Health”, University of Pisa, via del Borghetto 80, 56124 Pisa, Italy; andrea.serra@unipi.it; 4 Department of Agriculture Food and Environment, University of Pisa, Via del Borghetto 80, 56125 Pisa, Italy; 5 Department of Agri-Food Production and Environmental Sciences, University of Florence, via delle Cascine 5, 50144 Firenze, Italy; giuliana.parisi@unifi.it (G.P.); isabella.tucciarone@unifi.it (I.T.); 6 Department of Grain and Compound Feeds Technologies, Odesa National University of Technology, Kanatnaya 112, 65039 Odesa, Ukraine; fihurska@gmail.com; 7 Institute for Global Food Security, School of Biological Sciences, Queen’s University, BT9 5DL Belfast, UK; m.p.carey@qub.ac.uk (M.C.); katrina.campbell@qub.ac.uk (K.C.)

**Keywords:** aquaculture, alternative protein, insects, gilthead seabream, black soldier fly

## Abstract

The aquaculture sector has seen significant growth recently but also faces sustainability challenges due to the use of fish meal and fish oil. This project explored the potential of using partially defatted Hermetia illucens (black soldier fly) larvae meal (BSFL meal) as a sustainable alternative to fish meal in Sparus aurata diets. The trial was conducted with 132 fish reared in six tanks and fed two aquafeeds: a standard (CTRL) and an experimental (IF) in which fish meal was replaced (10%) with partially defatted BSFL meal. Fillets were analysed for chemical composition, elemental composition, fatty acid (FA) profile, primary and secondary oxidative products, cholesterol, pH and colour. Moreover, a panel of food experts (n = 26) was involved in a discrimination sensory test (duo–trio test) on raw and cooked fillets. The results showed no differences in chemical and physical analyses. The FA profile of IF was characterised by a significantly higher content of lauric acid (*p*-value 0.009) and myristic acid (*p*-value 0.049) than the CTRL ones. The panellists correctly identified the raw samples and found differences. On the other hand, only about 50% of the assessors correctly identified the cooked samples. The overall results suggest that partially defatted BSFL meal may represent a valid alternative for aquafeed production that could affect the sensory properties of raw fillets without altering their nutritional composition.

## 1. Introduction

Aquaculture is a fast-growing sector with significant implications for the economy, society and environmental sustainability [1]. According to the FAO 2024 report, global aquaculture production in 2022 reached a record of 130.9 million tonnes; 57% of the products were for direct human consumption, with a total value of USD 313 billion, representing an increase of 7.6% compared to 2020 [2]. Aquafeeds designed for carnivorous species primarily include fish meal (FM) and fish oil (FO), which are mainly extracted from small pelagic forage fish [3] and, to a lesser extent, but with an increasing trend, fish by-products are also used to produce fish meal and oil [4]. In response to the main challenge of not burdening natural fish stocks and producing sustainable feeds that ensure welfare, health and good performance, research has focused on exploring alternative protein sources that can reduce dependence on FM [5,6]. Many plant proteins have already been tested as alternatives for fish feed and some of these are already used as components of aquaculture feed [7]. Plant alternatives have the advantages of being widely available on the market and having affordable prices; however, they do not enable the complete replacement of fish meal in the case of carnivorous fish species because they have a high-carbohydrate content, different biological value in terms of amino acids, and can contain antinutritional factors [8,9,10]. In addition to plant proteins, scientific research has extended to a wide range of other alternative protein sources to replace FM, such as bone meal and meat, blood meal and poultry by-product flour and single-cell proteins [11]. With the Regulation (EU) 2017/893, the use of Processed Animal Proteins (PAPs) derived from insects was authorised for aquafeed formulation. In September 2021, the Reg. (EU) 2021/1372 extended the feed ingredient authorisation to pigs and poultry, and in November 2021, the Reg. (EU) 2021/1925 added silkworm (*Bombyx mori*) to the list of authorised insect species for the PAPs intended for the manufacturing of feed for farmed animals. Furthermore, in 2024, the European Commission’s Standing Committee on Plants, Animals, Food and Feed (PAFF Committee) legally clarified and recognised in the EU the use of live insects as feed for the above-mentioned species. Insects, particularly the black soldier fly (*Hermetia illucens*) larvae (BSFL), are seen as promising solutions for the near future due to their ability to grow on a wide variety of organic substrates and their potential for large-scale production. These characteristics make them a suitable circular economy model, which could contribute to the reduction in food waste and the production of protein-rich biomass for animal feed [12,13,14]. Insects can offer products with a nutritional profile comparable to that of fish meal but with a much lower environmental impact [15]. BSFL’s nutritional profile is strongly influenced by the substrate composition utilised for farming them, not only in protein content (ranging from 37 to 63% dry matter; DM) but also in fat content (ranging from 7 to 39% DM) [16]. BSFL are rich in saturated fatty acids, in particular, lauric acid, but their fatty acid profile can also be changed through diet [17,18]. Moreover, BSFL can provide adequate levels of essential amino acids, minerals and vitamins [19,20]. Recently, several authors tested the effects of aquafeeds containing partial substitution at different percentages and in different forms with *Hermetia illucens* larvae meals. Notably, Hua [21] reported that it is very important to keep balanced feed when insect meal is used in substitution of fish meal. Insect meals in balanced diets did not show negative effects on fish growth rate up to a certain level, up to 29% (±3%) dietary inclusion levels of BSFL. Production and trade data about gilthead seabream show that seabream production and consumption are mainly concentrated in the Mediterranean area [6,22,23]. Despite the use of insects as a feed ingredient that has been authorised by EU legislation and is technically feasible, the acceptance of this innovative feed by stakeholders and consumers—along with the fish products derived from it—is only beginning to be explored [24]. Several studies have reported minimal impacts on fillet quality from fish-fed BSFL at different percentages and in different forms, compared to standard-fed fish [25,26,27,28]. The literature about feeding gilthead seabream with BSFL-based feed reported that, principally, the fatty acid profile of the fillets was affected by the insect meal [26,27,28], showing an increase in saturated fatty acids (SFAs), particularly lauric acid, while eicosapentaenoic acid (EPA) and docosahexaenoic acid (DHA) contents were substantially not affected [26,27,28]. Pulido et al. [26] reported that a fish meal substitution of up to 75% with BSFL meal did not affect the commercially relevant physical characteristics of the fillet. Similarly, Moutinho et al. [28], using different inclusions of BSF oil, did not find significant differences in skin and fillet colours as well as in fillet textures. Interestingly, Busti et al. [27] showed that inclusion of BSFL meal of up to 15% did not negatively impact the fillets’ taste and quality. This study aimed to investigate the effects of a partial replacement of FM in the feed of gilthead seabream (*Sparus aurata*) on the quality characteristics and sensory properties of raw and cooked fillets, as evaluated by a panel of experts of the food service sector.

## 2. Materials and Methods

### 2.1. Ethical Statement

The care and handling of fish complied with the guidelines of ARRIVE and met the provisions of Directive 2010/63 of the Council of the European Union, which was recognised and adopted by the Italian government (DLgs No. 26/2014). The protocol was approved by the Ethics Committee for Animal Welfare and Use of the University of Pisa (No. 43/2024 B290E.N.51V, approval date: 11 June 2024).

### 2.2. Diet Formulation

Two isoenergetic, isonitrogenous, and isolipidic aquafeeds, formulated to meet the nutritional needs of *Sparus aurata*, were purchased from VRM Naturalleva srl (Verona, Italy): a standard diet containing 22.8% FM as fed basis (control, CTRL), and an experimental diet (IF) containing 10% BSFL meal partially defatted, replacing about 40% of the FM of the control feed (Table 1). This percentage of BSFL meal represents a cost-effective and tolerable fish meal substitution for *Sparus aurata* [29,30].

### 2.3. Fish Feeding Trial

The feeding trial was conducted in the RAS (Recirculating Aquaculture System) located at the Department of Agricultural, Food and Agro-Environmental Sciences of the University of Pisa, Pisa (Italy). One hundred and thirty-two *Sparus aurata* specimens kindly supplied by COSA Società Agricola a r.l, were used. Fish with an average weight of 364.09 g were randomly divided equally into 6 tanks (420 L each, for a stocking density of 19.1 kg/m^3^). Water flow was set to allow 7 renewals per day (122 L/h); the temperature was 23.4 ± 0.8 °C, with dissolved oxygen 5.3 ± 0.21 ppm and pH 7.3 ± 0.5. The salinity was measured by a salt refractometer and maintained at 30 ± 1.4 ppm. Also, NH_3_ (≤1 mg/L), NO_2_^−^ (≤0.25 mg/L) and NO_3_^−^ (≤50 mg/L) were monitored spectrophotometrically on a daily basis (Spectroquant ammonium test, Spectroquant nitrate test and Spectroquant nitrites test, Merck, Lab business, Darmstadt, Germany). Fish groups were left to adapt to the farming conditions over ten days before the tank was assigned to a diet (3 tanks per diet). Fish were fed till satiation once a day (at 9:00 am), 7 days a week for 2 months and the quantity of feed supplied to each tank was recorded. Photoperiod followed natural changes according to the season of the year (June–August; latitude: 43°42′42″48 N). At the end of the trial, fish were fasted for 24 h, before slaughtering. Any criteria for including or excluding animals were used.

### 2.4. Physical and Chemical Analyses

Five fish per tank were employed for the physical and chemical analyses. Both physical and chemical analyses were performed on the left sides of the fish. The tank was considered the experimental unit, while individual fish represented the observational units. Five fish per tank were employed for the physical and chemical analyses that were performed on the left fish sides. The colour of the skin and fillets was measured in triplicates in three anatomical points (cranial, medial and caudal) and expressed as lightness (L*), redness (a*) and yellowness (b*) indexes according to the CIElab system [31]. Colour parameters were measured using a Minolta CR300 Chroma meter (Minolta, Osaka, Japan), with an aperture size of 8 mm, Illuminant D65, and an incidence angle of 0°. The colourimeter was calibrated with a white tile (L* = 98.14, a* = −0.23 and b* = 1.89). The numerical total colour difference (ΔE) between the experimental groups at the level of skin and fillets was calculated as follows:

$${\Delta E}_{\beta -\alpha }={{[({L*}_{\;\beta }-{L*}_{\alpha })}^{2}+{\left({a*}_{\beta }\;-\;{a*}_{\alpha }\right)}^{2\;}+{\left({b*}_{\beta }-\;{b*}_{\alpha }\right)}^{2}]}^{0.5}\;$$

where L*_α_, a*_α_, b*_α_, and L*_β_, a*_β_, b*_β_ are, respectively, the values of IF and CTRL. A colour variation (ΔE) equal to 2.3 units corresponds to a just-noticeable difference (JND) for the human eye while a higher variation is considered discernible [32]. The pH values were measured in three fillet anatomical points (cranial, medial and caudal) using a pH metre (Eutech pH2700 Meter; Eutech Instruments Pte Ltd., Singapore) equipped with an insertion probe XS Sensor Standard S7 (XS Sensor, Modena, Italy) and an automatic temperature compensator. The pH metre was calibrated with buffer solutions at pH 4.01 and 7.01 (HI7004L and HI7007L; Hanna Instruments, Padova, Italy). Proximate composition analysis was performed on skinless fillets. Dry matter (DM) was determined by heating the sample at 105 °C till constant weight. The ether extract (EE) was assessed through a Soxtec extraction system (Tecator Soxtec System HT 1043; Foss Italia S.r.l, Padova, Italy). Crude protein (CP) was measured as total nitrogen using the Kjeldahl method and a nitrogen-to-protein conversion factor of 6.25 was used. Ash (ASH) content was analysed by incinerating the samples in a muffle oven at 550 °C and then the weight was determined.

### 2.5. X-Ray Fluorescence (XRF) Spectroscopy Analysis

The elemental concentration was determined on dried samples of fish fillets as previously described by Lim et al. [33]. In brief, the dried fish fillets were finely ground using a Retsch Planetary Ball mill PM100 with ceramic grinding balls and zirconium oxide grinding jars before being placed inside the XRF sample cups (Elementec, Maynooth, Ireland). For each sample, 2.5–3.0 g of finely ground powder was placed inside two 32 mm double open-ended XRF sample cups and covered on one side with a Prolene thin film (Chemplex Industries, Palm City, FL, USA). The samples were compressed at 200 psi for 25 s to a depth of ca. 4 mm. The depth and weight of each of the samples were recorded. The elements contained in the samples were analysed using an EDXRF spectrometer (Rigaku Nex CG benchtop XRF, Austin, TX, USA). The instrument was calibrated using the Fundamental Parameter method, and element measurements were performed under helium purging to improve the sensitivity for Mg and P. To determine the accuracy and reproducibility of the measurements, a certified reference material (CRM-RM8414) was used. The concentration of each element was calculated from the XRF spectra by the Rigaku RPF-SQX (“profile fitting–spectra quant X”) software [34]. Only elements present in the CRM and with good analytical recoveries were used.

### 2.6. Fatty Acid Profile and Fillet Oxidation Products

Lipids were extracted following the method described by Folch et al. [35], using 2 g of finely chopped fillet per sample. The total lipid content was then determined gravimetrically and reported as grams of lipid per 100 g of fillet. To analyse the fatty acid composition, a base-catalysed transesterification process was performed on 4 mg of total lipids to produce fatty acid methyl esters (FAMEs) [36]. These FAMEs were subsequently analysed using a Varian gas chromatograph (Varian Inc., Palo Alto, CA, USA) equipped with a flame ionisation detector (FID) and a Supelco OmegawaxTM 320 capillary column (30 m, 0.32 mm i.d., 0.25 μm film, polyethylene glycol-bonded phase; Supelco, Bellefonte, PA, USA). The injector and detector temperatures were set to 220 °C and 300 °C, respectively, with helium as the carrier gas at a constant flow rate of 1.5 mL/min. Chromatographic data were recorded using the Galaxie Chromatography Data System 1.9.302.952 (Varian Inc., Palo Alto, CA, USA). Fatty acids were identified by comparing FAME retention times to those of a Supelco 37-component FAME standard mix (Supelco, Bellefonte, PA, USA) and quantified using calibration curves, with tricosanoic acid (C23:0) (Supelco, Bellefonte, PA, USA) serving as an internal standard. Results were expressed as percentages of the total FAMEs. The fatty acid quality indexes, i.e., atherogenicity index (AI), thrombogenicity index (TI) [37], the ratio of hypocholesterolemic to hypercholesterolemic (h/H) [38] index and the Healthy Fatty Index (HFI) [39], were calculated as follows:

AI=[C12:0+(4×C14:0)+C16:0]/(ΣPUFAn_3+ΣPUFAn_6+ΣMUFA)]

TI=(C14:0+C16:0+C18:0)/[(0.5×ΣMUFA)+(0.5×ΣPUFAn_6)+(3×ΣPUFAn_3)+(ΣPUFAn_3/ΣPUFAn_6)]

h/H=(C18:1n_9+C18:2n_6+C20:4n_6+C18:3n_3+C20:5n_3+C22:5n_3+C22:6n_3)/(C14:0+C16:0)

HFI=[(ΣMUFA×2)+(ΣPUFAn_6×4)+(ΣPUFAn_3×8)+(ΣPUFAn_3/ΣPUFAn_6]/[(ΣSFA×1)+(ΣMUFA×0.5)+(ΣPUFAn_6×0.25)+(ΣPUFAn_3×0.125)+(ΣPUFAn_6/ΣPUFAn_3)]

To determine the cholesterol content in the fillets, 0.2 mL of lipid extract was mixed with 0.5 mL of 5α-cholestane (0.2 mg/mL in chloroform) (Supelco) as an internal standard. After evaporating the solvent, 5 mL of potassium hydroxide (0.5 M in methanol) was added, and the mixture was heated in a water bath at 95 °C for 40 min to facilitate lipid saponification. Subsequently, 4 mL of distilled water and 2 mL of n-hexane were introduced, and the upper phase was collected for gas chromatography analysis. The analysis was performed using a Varian GC 430 gas chromatograph (Varian Inc.), fitted with a flame ionisation detector and a Supelco SACTM fused silica capillary column (30 m × 0.25 mm i.d., 0.25-μm film; Supelco), sourced from Agilent Technologies (Santa Clara, CA, USA). A total volume of 1 μL of sample was injected with a 1:100 split ratio at 300 °C. The oven temperature was programmed to increase from 130 to 290 °C at a rate of 20 °C per minute over 8 min, then maintained at 290 °C for 11 min. The detector temperature was set at 300 °C, with helium as the carrier gas at a steady flow rate of 1.3 mL/min. To assess the lipid oxidation of the fillets, both primary and secondary oxidation products were measured as conjugated dienes (CDs) and thiobarbituric acid reactive substances (TBARSs), respectively. CDs were quantified using a spectrophotometric method, in which 0.1 μL of lipid extract was dissolved in 3 mL of pure hexane. The absorbance was then recorded at 232 nm using a Lambda EZ 150 UV/VIS spectrophotometer (Perkin Elmer, Waltham, MA, USA). The concentration of conjugated dienes was calculated using a molar extinction coefficient of 25,200 mL/mmol, with results expressed as mmol Hp per 100 g of the sample. To assess the TBARSs, 2 g of homogenised fillet samples were analysed following the spectrophotometric method described by Vyncke [40]. This method determines TBARSs by quantifying TBARSs in terms of malondialdehyde equivalents (MDA eq.). Absorbance readings were taken at 532 nm using the same spectrophotometer, and the results were expressed as mg MDA eq. per 100 g of fresh sample.

### 2.7. Sensory Analysis

A discrimination duo–trio test [41] was conducted on both raw and cooked seabream fillets, using rectangular portions (2 × 2 cm) from the epaxial part of the skinless fillet (approved by the Institutional Ethics Committee of the University of Parma (protocol number 0145716 and date of approval 11 June 2024)). The test took place at the School of Italian Culinary Arts ALMA (Colorno, Parma, Italy) and involved 26 trained panellists who evaluated both the raw and cooked samples. For the raw samples, each respondent was presented with a neutral-coloured plastic plate containing three coded portions, one of which was a constant reference (CTRL) and two others representing the two different diets (CTRL vs. IF). Panellists were instructed to identify the most different sample from the reference and then answer an open-ended questionnaire (e.g., “please briefly explain your selection”) to further understand the sensory differences noted by the panellists concerning the fillets. A similar serving procedure was followed for the cooked fillet samples; each respondent was presented with a neutral-coloured plastic plate containing three coded portions, but in this case, the IF samples were used as the reference. Once again, panellists were instructed to indicate the most different sample from the reference by filling out an open-ended questionnaire (Supplementary Materials). The cooked fillets were prepared by cooking them vacuum-sealed in a temperature-controlled water bath at 62 °C for 20 min. All panellists gave informed consent prior to their participation in the sensory analysis. Examples of the duo–trio tests are reported in the Supplementary Materials (Table S1).

### 2.8. Statistical Analysis

Statistical analysis of the data was conducted using the software JMP^®^, PRO 18 [42]. The tank was used as the experimental unit. A Student *t*-test for independent samples was conducted in order to identify significant differences (*p*-value < 0.05) in physical and chemical analysis data between fish fed the two diets (control standard feed, CTRL, and experimental diet containing 10% of black soldier fly larvae meal, IF). In addition, for the duo–trio test, the number of correct responses was compared to the critical threshold for statistical significance based on various probability levels (i.e., the minimum number of correct answers considered necessary to confirm a detectable difference) [41]. This approach allowed us to determine whether the panellists could reliably distinguish between the reference and the test samples. A significance level of α = 0.05 was used to assess whether the number of correct responses exceeded the critical threshold for statistical significance. For open-ended questions, responses were coded based on common themes and topics to identify key aspects mentioned by the panellists.

## 3. Results and Discussion

### 3.1. Proximate and Physico-Chemical Analyses

The proximate composition analysis showed no significant differences between gilthead seabream fed the CTRL and IF diets (Table 2). According to the data reported by Anedda et al. [43] and Busti et al. [27], who conducted trials substituting 10% of FM with *Hermetia illucens* meal in feeds for gilthead seabream, there were no significant differences in the proximate composition of fish fillets.

On the other hand, previous research observed changes in the physical and chemical characteristics of the skin and fillets when insects were employed as substitutes for FM in aquaculture feeds. These modifications could mainly be attributable to the amount of substitution of the fish meal with insect meal and the defatting degree of the meal (partially/fully defatted or full-fat), as reported by Karapanagiotidis et al. [44], and also in relation to fish species considered. Colour measurements of the skin and fillets did not show any significant difference between the dietary treatments (Table 3). Colour is an essential parameter to consider in terms of quality because the appearance of skin and fillets is one of the main quality indicators for consumers when buying fish [45,46]. Although there were no statistically significant differences in skin colour measurements between the dietary treatments, the total colour difference (ΔE) was 2.48. As reported above, the minimum ΔE value for the human eye to perceive a noticeable difference is 2.3. This suggests a slight difference, mainly attributable to the higher lightness (L*) and yellowness (b*) values observed in the skin of IF fish, along with a lower redness index (a*) in the CTRL fish (Table 3). The fillets’ ΔE resulted in 0.45, highlighting a lack of noticeable difference by the human eyes in colour between the two dietary treatments. As reported by Pulcini et al. [47], diets containing insect meal had a mild colouring capacity on the skin pigmentation of gilthead seabream. The same was observed for the pH values of fish fillets, confirming that the inclusion of 10% of BSFL meal did not cause pH variation in the fillets. These results corroborate what was reported from other authors who tested BSFL meal as an FM substitute and confirmed that the *post-mortem* acidification process in fillet muscle is not influenced [27,48]. Moutinho et al. [28], replacing vegetable oil with *Hermetia illucens* oil (42%, 84%, and 100%), reported lower pH values than fillets from fish fed a standard aquafeed. However, it is necessary to underline that pH values could be affected by the *rigor mortis* resolution, so differences between studies could also be related to the time of measurement of the pH [49].

### 3.2. X-Ray Fluorescence (XRF) Spectroscopy

The results of the XRF analysis are reported in Table 4. Fish may accumulate elements depending on the environment and dietary intake. The mineral content in fish is fundamental for several biological processes such as enzyme cofactors and activators, pH regulation, osmotic pressure maintenance, skeletal muscle formation and transmission of electric impulses in the nervous system [50]. Furthermore, the elemental composition of fish fillets, like that of other animal tissues, is a relevant safety issue for consumers [51]. The XRF results showed that CTRL fillets were richer in phosphorus (P) than IF fillets. The elevated P levels in IF fillets may be attributed to a higher dietary phosphorus intake or defatting process, which affects phospholipid content. In contrast, Oteri et al. [52], using BSFL meal as a partial replacement for fish meal in the gilthead seabream diet (35% and 50%), reported a significantly lower value of P in fish fed the control feed. At the same time, IF fillets resulted in higher Ca levels than CTRL fillets. Consequently, the Ca/P ratio, an important value for the absorption of other minerals [52], resulted in higher IF than CTRL fillets (0.09 vs. 0.06). Mg, S and Zn were significantly higher in CTRL than in IF fillets.

### 3.3. Fatty Acid Profile of Fillets

The fatty acid profile of the gilthead seabream fillets is summarised in Table 5. The main fatty acids in fillets derived from both the dietary treatments were oleic acid (C18:1), linoleic acid (C18:2n-6) and palmitic acid (C16:0), representing about 70% of the total fatty acid composition (Table 5). The diet did not affect the main fatty acids, but significant differences between treatments were identified for lauric (C12:0), myristic (C14:0) and stearic acids (C18:0, Table 5). IF fillets showed higher levels of C12:0 and C14:0, while C18:0 was higher in CTRL fillets. This variation could be ascribable to the fatty acid compositions of BSFL meal, since the meal was not completely defatted. Indeed, lauric acid is the most abundant fatty acid in black soldier fly larvae [53] and the IF diet had 16 times the content of this fatty acid in comparison with the CTRL diet (Table 1). Our results are in line with the study of Busti et al. [27] in which they showed that the content of lauric acid in gilthead seabream fillets increased with high levels of BSFL meal inclusion. Even if myristic acid in IF and CTRL diets do not differ largely (Table 1), the significant variation in the fillets could be related to the BSFL meal. Likewise, Anedda et al. [43] reported a small but significant effect on the lauric and myristic acid content of gilthead seabream fillets when the FM was replaced by BSFL meal at 10%. It seems that the statistically significant difference in stearic acid content between CTRL and IF cannot be attributed to the diet effect as the two diets showed similar C18:0 contents. In general, the high SFA content in BSFL is reflected in the muscle tissue of fish that consume this ingredient [54]. However, in this case, even if the employed BSFL meal was partially defatted, it did not significantly affect the total SFA content in the fish (Table 5). Oleic acid (C18:1) was found to be the most abundant MUFA both in CTRL and IF fillets, and this result can be attributable to the dietary composition (Table 1). Similar findings were reported by Busti et al. [27] and Anedda et al. [43], who tested diets that include 10% of BSFL meal in gilthead seabream. The fatty acid analysis results showed that linoleic acid was the most abundant polyunsaturated fatty acid (PUFA), followed by α-linolenic acid (C18:3n-3). Incorporating BSFL meal into the feed did not significantly alter the PUFA content of the diet in comparison to the CTRL one (Table 1); therefore, no variation for this group of fatty acids was expected in the fatty acid profile of fillets. Similarly, Bruni et al. [55] reported that the inclusion of a BSFL meal in rainbow trout (*Oncorhynchus mykiss*) did not impair the PUFA content. In contrast, PUFA content might be altered when including partially defatted BSFL meal in fish feed. Mancini et al. [56] and Caimi et al. [57] showed that including partially defatted BSFL meal in the diets of rainbow trout led to a significant depletion of the PUFA content of the fillets. Also, Belghit et al. [58] reported that the inclusion of *Hermetia illucens* oil into the feed of Atlantic salmon (*Salmo salar*) resulted in an increase in SFAs and a decrease in MUFAs and PUFAn-3 in fish. When evaluating the potential of a new ingredient for aquafeed formulation, such as insects, it is not only essential to ensure optimal fish growth but also to maintain the quality of fish for consumers. Consumers generally have a positive perception of fish, viewing it as a healthy dietary choice. Fish is indeed considered an essential part of human nutrition, providing important nutrients such as n-3PUFA fatty acids [59]. Fish are unable to synthesise PUFA n-3, so they must acquire them through the diet. As a result, we obtained no significant differences in the content of eicosapentaenoic acid (C20:5n-3, EPA) and docosahexaenoic acid (C22:6n-3, DHA) of the gilthead seabream fillets fed the two diets (Table 5). These results were expected as the diets did not differ in EPA and DHA percentages, and typically *Hermetia illucens* larvae do not contain these acids [60]. These results suggested that gilthead seabream could be fed in the last rearing period—till the standard size is achieved—with a BSFL meal substitution without impairing the content of these essential FAs in fillets. According to the FAO [61] report and EFSA [62] scientific opinion, the adequate daily intake of EPA + DHA should reach at least 250 mg for healthy adults. CTRL and IF fillets can both provide >250 mg of EPA + DHA per serving size (100 g). The minimum recommended amount of EPA + DHA correspond to 1750 mg per week, meaning that 33.9 g or 33.6 g of raw CTRL or IF per week can supply that amount of EPA + DHA. As no variations in PUFAn-3 and PUFAn-6 percentages were revealed in relation to the diet, the ratio of n-3/n-6 was also unaffected. Our results only showed a slightly lower value for IF, but the difference from the CTRL group was not significant.

As the main fatty acids were not modified, the fatty acid quality indexes did not differ significantly between CTRL and IF fillets (Table 5). The atherogenic index (AI) and thrombogenicity index (TI) were calculated to take into consideration the relationship between pro- and anti-thrombogenic FAs; therefore, values <0.5 for AI and <1.0 for TI are recommended to prevent cardiovascular disorders [38,62]. In the study conducted by Mouthinho et al. [28], juvenile gilthead seabreams were fed diets containing different percentages of *Hermetia illucens* oil. The results showed a negative effect on AI and TI indexes. This could be attributable to the low content of PUFAs and high content of SFAs in *Hermetia illucens* oil. Similar worsening effects on the lipids’ health indexes were previously reported by other authors when a partially defatted and full-fat BSFL meal was included in the feed of rainbow trout [48,55]. Evaluating the effects of insect feeding on three fish species (*Sparus aurata*, *Tinca tinca*, *Oncorhynchus mykiss*), Fabrikov et al. [54] reported that insect-containing diets worsened the AI and TI of fillets. The h/H ratio is utilised to assess the nutritional and health-promoting properties of a food product, particularly regarding the influence of fatty acids on cholesterol metabolism. Higher h/H values are generally seen as more beneficial for human health [63,64,65]. As shown in Table 5, the h/H values of CTRL and IF groups were similar. The Healthy Fatty Index (HFI) is calculated by differentiating the various classes of FAs but also considering the different classes of FAs and their role in cardiovascular diseases [40]. The HFI, in contrast to the AI and TI, is a direct index because the higher values correspond to healthier foods. The CTRL fillets exhibited slightly higher HFI values than IF (Table 5). The cholesterol content did not show a significant difference (*p* = 0.945) between the treatments, with values of 62.82 ± 7.14 mg/100 g in the CTRL and 62.56 ± 4.41 mg/100 g in the IF fillets. These findings are consistent with those reported by Orban et al. [66], which described values of 72.89 ± 10.85 mg/100 g in farmed gilthead seabream fillets. The primary oxidation products measured as conjugated dienes were not significantly affected by the dietary treatments (CTRL: 0.166 ± 0.03 mol/kg muscle; IF: 0.173 ± 0.02 mol/kg muscle; *p* = 0.681); likewise, the secondary oxidation products, measured as TBARSs, did not result in being statistically different (0.025 ± 0.00 mg MDA/kg and 0.173 ± 0.06 mg MDA/kg for CTRL and IF, respectively; *p* = 0.330). These results suggest that replacing 10% of FM with partially defatted BSFL meal did not influence the oxidation of the fillets’ lipid fraction. The results of the oxidation products may be attributable to the similar total SFA and PUFA contents of the fillets. Similar findings were reported by Busti et al. [27] using a BSFL meal as a 10% FM substitution. Moutinho et al. [28] conducted a study employing *Hermetia illucens* oil inclusions as a partial replacement for vegetable oils in gilthead seabream feed and reported a significant reduction in lipid peroxidation level (TBARSs) as well as an increase in conjugated dienes. This was related to the fatty acid profile of fillets associated with *Hermetia illucens* oil inclusions. A reduction in TBARSs was also reported in rainbow trout fed with full-fat BSFL meal [55].

### 3.4. Sensory Analysis

Results from the duo–trio test showed that out of 26 panellists, 23 correctly identified the raw fillet samples that differed from the reference. The differences in the raw samples were perceived as significantly different at the 95% confidence level (α = 0.05). Additionally, with 22 correct responses, the samples were considered significantly different at a 99% confidence level (α = 0.01). Open-ended responses used to explore the sensory differences noted by the panellists in relation to the fillets showed that the main differences were related to colour, texture, flaking and odour intensity. Interestingly, even if the ∆E of fillets was lower than the threshold of perception by the human eye (0.45, while the threshold is set at 2.3), the reason behind the panellists’ decisions extended beyond solely the colour. Indeed, in open-ended responses, the perception of the panellists was a mix of visual attributes that were also related to the structure and odour of the fillets. These two elements were surely also related to the fatty acid minerals profiles. In contrast, among the cooked fillet samples, only 14 assessors correctly identified the sample that differed from the reference. Since the number of correct responses required to conclude a significant difference at α = 0.05 is 18, we can conclude that incorporating insects into the fish diet affects the characteristics of the raw fillets but does not significantly impact the sensory properties of the cooked product. Notably, the fillets were cooked vacuum-sealed in a temperature-controlled water bath at 62 °C (20 min) in order to minimise the effect of the cooking on the sensory attributes. Other cooking techniques or the use of seasoning might have different effects. Our findings are in line with previous studies evaluating the impact of *Hermetia illucens* larvae meal inclusion on fish fillet quality. Busti et al. [27], for instance, reported that the inclusion of defatted *Hermetia illucens* larvae meal did not induce off-flavours in seabream fillets, with no significant differences in appearance, mouthfeel and texture between control and experimental diets. Similarly, other studies on various fish species have also shown that most sensory attributes remain unaffected by the inclusion of *Hermetia illucens* as a substitute for fish meal [58,67,68,69]. However, it is worth noting that most of these studies have primarily focused on the sensory characteristics of cooked fish, whereas fewer works have investigated potential differences in raw fillets. The present study highlights that while insect meal inclusion does not significantly alter the sensory properties of cooked fillets, differences can be perceived in the raw product. This suggests that further research should explore the implications of these raw fillet differences, particularly in relation to consumer perception and processing properties.

## 4. Conclusions

Our study provided a characterisation of gilthead seabream fed with a 10% replacement of fish meal with partially defatted black soldier fly meal. We examined the effects on the chemical composition of fillets and their quality. Our results showed that a substitution of the FM with partially defatted BSFL meal did not significantly impact the proximate composition, colour and pH of fillets. Gilthead seabream fed 10% of partially defatted BSFL meal contains lauric and myristic acids due to the fatty acid composition of the larvae. However, this does not cause significant changes in the fatty acid composition of fillets and, in particular, in the EPA and DHA content. The overall results suggest that partially defatted BSFL meal may represent a valid FM alternative for aquafeed production that could affect the sensory properties of raw fillets without altering the nutritional composition.

## Figures and Tables

**Table 1:** Diets (ingredients %) and proximate composition (% as fed).

Ingredients	CTRL	IF
Fish meal	22.8	14.1
Black soldier fly larvae meal	0.0	10.0
Fish oil (trimming by-product)	1.2	2.8
Fish oil (whole-fish bycatch)	9.7	7.9
Soybean meal	13.2	13.2
Guar meal	10.5	10.6
Wheat flour	7.9	5.4
Maize gluten meal	26.4	26.4
Wheat gluten meal	4.9	5.7
DL-methionine	0.3	0.3
Emulsifier (E484)	0.2	0.2
Monoammonium phosphate	0.8	1.2
HCl lysine	0.3	0.4
Premix vitamins and minerals	0.5	0.5
Taurine	0.2	0.3
Hydrolyzed shrimp protein (liquid)	1.0	1.0
Rovimix^®^ Stay C 35%	0.1	0.1
Proximate composition (% as fed)		
Crude fat	15.8	15.8
Crude protein	49.2	49.2
Fibre	1.4	2.4
Ash	5.8	5.4
Gross energy (MJ/kg)	19.4	19.1
Fatty acid composition (% of total fatty acids)		
C12:0	0.1	1.8
C14:0	1.8	2.1
C18:0	3.2	3.1
ƩSFA	18.5	20.5
C18:1	40.5	39.3
ƩMUFA	46.5	45.3
C18:3n-3	5.0	4.9
C20:5n-3	2.1	2.0
C22:6n-3	3.5	3.2
ƩPUFAn-3	12.1	11.7
C18:2n-6 cis	21.2	20.8
ƩPUFAn-6	22.3	21.9
ƩPUFA	35.1	34.3

CTRL: Standard diet; IF: experimental diet containing 10% of black soldier fly larvae meal; fish meal (FM): protein content 66%; vitamin and mineral premix (kg of product): vitamin A 7.893 UI, vitamin D3 2.105 UI, vitamin C 246 mg, vitamin E 316 mg, Cu 5 mg, I 2 mg, Fe 210 mg, Mn 21 mg, Se 0.2 mg, and Zn 42 mg.

**Table 2:** Proximate composition (mean values) of fillets from gilthead seabreams fed the standard (CTRL) or experimental (IF) diet.

Proximate Composition	CTRL	IF	RMSE	*p*-Value
n	3	3		
Dry matter%	29.95	30.48	2.059	0.664
Ether extract (on dry matter %)	31.24	29.52	4.569	0.530
Crude protein (on dry matter %)	64.22	60.74	3.307	0.098
Ashes (on dry matter %)	4.47	4.60	0.181	0.267

CTRL: Standard diet; IF: experimental diet containing 10% of black soldier fly larvae meal; RMSE: root mean square error.

**Table 3:** Colour indexes and pH of skin and fillets (mean values) of gilthead seabreams fed the standard (CTRL) and experimental (IF) diets.

		CTRL	IF	RMSE	*p*-Value
n		3	3		
Skin					
	L*	71.36	73.80	6.938	0.158
	a*	0.72	0.49	0.559	0.100
	b*	5.61	6.02	2.090	0.428
Fillet					
	L*	44.63	44.19	5.223	0.736
	a*	1.78	1.84	0.949	0.803
	b*	−0.54	−0.64	1.079	0.700
	pH	7.35	7.34	0.055	0.616

CTRL: Standard diet; IF: experimental diet containing 10% of black soldier fly larvae meal; L*: lightness; a*: redness; b*: yellowness. RMSE: root mean square error.

**Table 4:** X-ray fluorescence spectroscopy analysis (mean values) of gilthead seabream fillets fed the standard (CTRL) and experimental (IF) diets (ppm on a DM basis).

Mineral	CTRL	IF	RMSE	*p*-Value
n	3	3		
Mg	777.90	647.53	59.341	0.004
Al	145.51	145.93	5.478	0.895
P	6704.21	5969.08	312.379	0.002
S	5925.39	5093.25	297.032	0.001
Cl	1391.65	1383.63	59.921	0.821
K	15117.90	14490.40	700.077	0.152
Ca	433.98	533.87	45.641	0.004
Fe	14.72	14.42	1.286	0.697
Cu	4.19	4.21	0.997	0.978
Zn	14.92	12.89	1.326	0.025

CTRL: Standard diet; IF: experimental diet containing 10% of black soldier fly larvae meal; RMSE: root mean square error.

**Table 5:** Fatty acid profile (% of total fatty acids, mean values) of fillets from gilthead seabreams fed the standard (CTRL) or experimental (IF) diets.

Fatty Acid	CTRL	IF	RMSE	*p*-Value
n	3	3		
C12:0	0.04	0.15	0.061	0.009
C14:0	1.43	1.54	0.083	0.049
C16:0	11.23	11.16	0.252	0.644
C18:0	2.81	2.61	0.121	0.017
C20:0	0.28	0.29	0.017	0.558
C22:0	0.15	0.14	0.016	0.442
ƩSFA	16.59	16.55	0.348	0.846
C16:1	3.15	3.30	0.189	0.207
C18:1	37.86	38.59	1.018	0.242
C20:1	2.32	2.37	0.097	0.425
C22:1	1.20	1.26	0.007	0.146
ƩMUFA	45.04	46.02	1.178	0.178
C18:3n-3	8.33	7.35	0.946	0.103
C18:4n-3	0.29	0.32	0.040	0.224
C20:5n-3	1.30	1.36	0.097	0.320
C22:6n-3	3.85	3.85	0.401	0.980
ƩPUFAn-3	15.39	14.45	1.040	0.151
C18:2n-6 cis	20.90	20.92	0.522	0.950
C18:3n-6	0.14	0.17	0.050	0.406
C20:2n-6	0.69	0.65	0.073	0.299
ƩPUFAn-6	22.54	22.51	0.515	0.945
ƩPUFA	38.37	37.43	1.355	0.255
Others	3.53	3.49	0.143	0.622
n-3/n-6	0.68	0.64	0.042	0.119
AI	0.20	0.21	0.007	0.219
TI	0.19	0.20	0.010	0.490
h/H	5.61	5.57	0.182	0.739
HFI	6.32	6.15	0.235	0.238

CTRL: Standard diet; IF: experimental diet containing 10% of black soldier fly larvae meal. The fatty acids C15:0, C17:0, C18:3n-4, C20:3n-6, C20:3n-3, C20:4n-3, C20:4n-6, C22:2n-6, C22:5n-6, C22:5n-3, and C24:1, detected at a percentage acid < 0.1, are not listed in the table but are included in the sums. n3/n6: Ratio PUFAn-3/PUFAn-6; AI: atherogenic index; TI: thrombogenicity index; h/H: hypocholesterolemic-to-hypercholesterolemic ratio; HFI: Healthy Fatty Index. RMSE: root mean square error.

## Data Availability

The original contributions presented in this study are included in the article/Supplementary Materials. Further inquiries can be directed to the corresponding author. The data that support the findings of this study are available from the corresponding author, upon reasonable request.

## Supplementary Materials

The following supporting information can be downloaded at: https://www.mdpi.com/article/10.3390/foods14173107/s1, Table S1: Duo-trio tests evaluation documents.

## References

[1] Bohnes F.A., Hauschild M.Z., Schlundt J., Nielsen M., Laurent A. (2022). Environmental Sustainability of Future Aquaculture Production: Analysis of Singaporean and Norwegian Policies. Aquaculture.

[2] FAO (2024). The State of World Fisheries and Aquaculture 2024—Blue Transformation in Action.

[3] FAO (2022). The State of World Fisheries and Aquaculture 2022. Towards Blue Transformation.

[4] Naylor R.L., Hardy R.W., Buschmann A.H., Bush S.R., Cao L., Klinger D.H., Little D.C., Lubchenco J., Shumway S.E., Troell M. (2021). A 20-Year Retrospective Review of Global Aquaculture. Nature.

[5] Aragão C., Gonçalves A.T., Costas B., Azeredo R., Xavier M.J., Engrola S. (2022). Alternative Proteins for Fish Diets: Implications beyond Growth. Animals.

[6] Porcino N., Genovese L. (2022). Review on Alternative Meals for Gilthead Seabream, Sparus Aurata. Aquac. Res..

[7] Yan Q., Zhu X., Yang Y., Han D., Xie S. (2014). Feasibility of Partial Replacement of Fishmeal with Proteins from Different Sources in Diets of Korean Rockfish (Sebastes Schlegeli). J. Ocean Univ. China.

[8] Hussain S.M., Bano A.A., Ali S., Rizwan M., Adrees M., Zahoor A.F., Sarker P.K., Hussain M., Arsalan M.Z.-H., Yong J.W.H. (2024). Substitution of Fishmeal: Highlights of Potential Plant Protein Sources for Aquaculture Sustainability. Heliyon.

[9] Serra V., Pastorelli G., Tedesco D.E.A., Turin L., Guerrini A. (2024). Alternative Protein Sources in Aquafeed: Current Scenario and Future Perspectives. Vet. Anim. Sci..

[10] Krogdahl Å., Penn M., Thorsen J., Refstie S., Bakke A.M. (2010). Important Antinutrients in Plant Feedstuffs for Aquaculture: An Update on Recent Findings Regarding Responses in Salmonids. Aquac. Res..

[11] Hua K., Cobcroft J.M., Cole A., Condon K., Jerry D.R., Mangott A., Praeger C., Vucko M.J., Zeng C., Zenger K. (2019). The Future of Aquatic Protein: Implications for Protein Sources in Aquaculture Diets. One Earth.

[12] Salomone R., Saija G., Mondello G., Giannetto A., Fasulo S., Savastano D. (2017). Environmental Impact of Food Waste Bioconversion by Insects: Application of Life Cycle Assessment to Process Using *Hermetia lllucens*. J. Clean. Prod..

[13] Smetana S., Schmitt E., Mathys A. (2019). Sustainable Use of *Hermetia lllucens* Insect Biomass for Feed and Food: Attributional and Consequential Life Cycle Assessment. Resour. Conserv. Recycl..

[14] Alfiko Y., Xie D., Astuti R.T., Wong J., Wang L. (2022). Insects as a Feed Ingredient for Fish Culture: Status and Trends. Aquac. Fish..

[15] Cappellozza S., Leonardi M.G., Savoldelli S., Carminati D., Rizzolo A., Cortellino G., Terova G., Moretto E., Badaile A., Concheri G. (2019). A First Attempt to Produce Proteins from Insects by Means of a Circular Economy. Animals.

[16] Barragan-Fonseca K.B., Dicke M., van Loon J.J.A. (2017). Nutritional Value of the Black Soldier Fly (*Hermetia lllucens* L.) and Its Suitability as Animal Feed—A Review. J. Insects Food Feed.

[17] Li S., Ji H., Zhang B., Tian J., Zhou J., Yu H. (2016). Influence of Black Soldier Fly (*Hermetia lllucens*) Larvae Oil on Growth Performance, Body Composition, Tissue Fatty Acid Composition and Lipid Deposition in Juvenile Jian Carp (*Cyprinus carpio* var. Jian). Aquaculture.

[18] Danieli P.P., Lussiana C., Gasco L., Amici A., Ronchi B. (2019). The Effects of Diet Formulation on the Yield, Proximate Composition, and Fatty Acid Profile of the Black Soldier Fly (*Hermetia lllucens* L.) Prepupae Intended for Animal Feed. Animals.

[19] Fuso A., Barbi S., Macavei L.I., Luparelli A.V., Maistrello L., Montorsi M., Sforza S., Caligiani A. (2021). Effect of the Rearing Substrate on Total Protein and Amino Acid Composition in Black Soldier Fly. Foods.

[20] Finke M.D. (2013). Complete Nutrient Content of Four Species of Feeder Insects. Zoo Biol..

[21] Hua K. (2021). A meta-analysis of the effects of replacing fish meals with insect meals on growth performance of fish. Aquaculture.

[22] Llorente I., Fernández-Polanco J., Baraibar-Diez E., Odriozola M.D., Bjørndal T., Asche F., Guillen J., Avdelas L., Nielsen R., Cozzolino M. (2020). Assessment of the Economic Performance of the Seabream and Seabass Aquaculture Industry in the European Union. Mar. Polic..

[23] Mhalhel K., Levanti M., Abbate F., Laurà R., Guerrera M.C., Aragona M., Porcino C., Briglia M., Germanà A., Montalbano G. (2023). Review on Gilthead Seabream (*Sparus aurata*) Aquaculture: Life Cycle, Growth, Aquaculture Practices and Challenges. J. Mar. Sci. Eng..

[24] Sogari G., Oddon S.B., Gasco L., van Huis A., Spranghers T., Mancini S. (2023). Review: Recent Advances in Insect-Based Feeds: From Animal Farming to the Acceptance of Consumers and Stakeholders. Animal.

[25] Secci G., Mancini S., Iaconisi V., Gasco L., Basto A., Parisi G. (2019). Can the Inclusion of Black Soldier Fly (*Hermetia lllucens*) in Diet Affect the Flesh Quality/Nutritional Traits of Rainbow Trout (*Oncorhynchus mykiss*) after Freezing and Cooking?. Int. J. Food Sci. Nutr..

[26] Pulido L., Secci G., Maricchiolo G., Gasco L., Gai F., Serra A., Conte G., Parisi G. (2022). Effect of Dietary Black Soldier Fly Larvae Meal on Fatty Acid Composition of Lipids and Sn-2 Position of Triglycerides of Marketable Size Gilthead Sea Bream Fillets. Aquaculture.

[27] Busti S., Magnani M., Badiani A., Silvi M., Baldi G., Soglia F., Petracci M., Sirri F., Gasco L., Brambilla F. (2023). Effect of Different Inclusion Levels of Defatted *Hermetia lllucens* Larvae Meal on Fillet Quality of Gilthead Sea Bream (*Sparus aurata*). J. Insects Food Feed.

[28] Moutinho S., Oliva-Teles A., Pulido-Rodríguez L., Parisi G., Magalhães R., Monroig Ó., Peres H. (2024). Effects of Black Soldier Fly (*Hermetia lllucens*) Larvae Oil on Fillet Quality and Nutritional Traits of Gilthead Seabream. Aquaculture.

[29] Randazzo B., Zarantoniello M., Cardinaletti G., Cerri R., Giorgini E., Belloni A., Contò M., Tibaldi E., Olivotto I. (2021). *Hermetia illucens* and poultry by-product meals as alternatives to plant protein sources in gilthead seabream (Sparus aurata) diet: A multidisciplinary study on fish gut status. Animals.

[30] Bosi A., Banfi D., Moroni F., Ceccotti C., Giron M.C., Antonini M., Giaroni C., Terova G. (2021). Effect of partial substitution of fishmeal with insect meal (*Hermetia illucens*) on gut neuromuscular function in Gilthead sea bream (Sparus aurata). Sci. Rep..

[31] (1976). Official Recommendations on Uniform Colour Spaces, Colour Differences Equations and Metric Colour Terms.

[32] Sharma G. (2003). Digital Color Imaging Handbook.

[33] Lim C.M., Carey M., Williams P.N., Koidis A. (2021). Rapid Classification of Commercial Teas According to Their Origin and Type Using Elemental Content with X-Ray Fluorescence (XRF) Spectroscopy. Curr. Res. Food Sci..

[34] Rigaku Soils Plant Mater. (1385). https://rigaku.com/products/xrf-spectrometers/edxrf/application-notes/edxrf1385-agricultural-soils-plant-materials.

[35] Folch J., Lees M., Stanley G.H.S. (1957). A Simple Method for the Isolation and Purification of Total Lipides from Animal Tissues. J. Biol. Chem..

[36] Christie W.W. (1982). A Simple Procedure for Rapid Transmethylation of Glycerolipids and Cholesteryl Esters. J. Lipid Res..

[37] Ulbricht T.L.V., Southgate D.A.T. (1991). Coronary Heart Disease: Seven Dietary Factors. Lancet.

[38] Fernandes C.E., Vasconcelos M.A.d.S., Ribeiro M.d.A., Sarubbo L.A., Andrade S.A.C., Filho A.B.d.M. (2014). Nutritional and Lipid Profiles in Marine Fish Species from Brazil. Food Chem..

[39] Dal Bosco A., Cavallo M., Menchetti L., Angelucci E., Mancinelli A.C., Vaudo G., Marconi S., Camilli E., Galli F., Castellini C. (2024). The Healthy Fatty Index Allows for Deeper Insights into the Lipid Composition of Foods of Animal Origin When Compared with the Atherogenic and Thrombogenicity Indexes. Foods.

[40] Vyncke W. (1970). Direct Determination of the Thiobarbituric Acid Value in Trichloracetic Acid Extracts of Fish as a Measure of Oxidative Rancidity. Fette Seifen Anstrichm..

[41] (2017). Sensory Analysis—Methodology—Duo-Trio Test.

[42] JMP® PRO 18.

[43] Anedda R., Melis R., Palomba A., Vitangeli I., Biosa G., Braca A., Antonini M., Moroni F., Rimoldi S., Terova G. (2023). Balanced Replacement of Fish Meal with *Hermetia lllucens* Meal Allows Efficient Hepatic Nutrient Metabolism and Increases Fillet Lipid Quality in Gilthead Sea Bream (*Sparus aurata*). Aquaculture.

[44] Karapanagiotidis I.T., Neofytou M.C., Asimaki A., Daskalopoulou E., Psofakis P., Mente E., Rumbos C.I., Athanassiou C.G. (2023). Fishmeal Replacement by Full-Fat and Defatted *Hermetia lllucens* Prepupae Meal in the Diet of Gilthead Seabream (*Sparus aurata*). Sustainability.

[45] Matos E., Dias J., Dinis M.T., Silva T.S. (2017). Sustainability vs. Quality in Gilthead Seabream (*Sparus aurata* L.) Farming: Are Trade-offs Inevitable?. Rev. Aquac..

[46] García de la Serrana D., Fontanillas R., Koppe W., Fernández-Borràs J., Blasco J., Martín-Pérez M., Navarro I., Gutiérrez J. (2013). Effects of Variable Protein and Lipid Proportion in Gilthead Sea Bream (*Sparus aurata*) Diets on Fillet Structure and Quality. Aquac. Nutr..

[47] Pulcini D., Capoccioni F., Franceschini S., Martinoli M., Tibaldi E. (2020). Skin Pigmentation in Gilthead Seabream (*Sparus aurata* L.) Fed Conventional and Novel Protein Sources in Diets Deprived of Fish Meal. Animals.

[48] Renna M., Schiavone A., Gai F., Dabbou S., Lussiana C., Malfatto V., Prearo M., Capucchio M.T., Biasato I., Biasibetti E. (2017). Evaluation of the Suitability of a Partially Defatted Black Soldier Fly (*Hermetia lllucens* L.) Larvae Meal as Ingredient for Rainbow Trout (Oncorhynchus Mykiss Walbaum) Diets. J. Anim. Sci. Biotechnol..

[49] El Rammouz R., Abboud J.S., Abboud M., Mur A.E., Yammine S., Jammal B.H. (2013). pH, Rigor Mortis and Physical Properties of Fillet in Fresh Water Fish: The Case of Rainbow Trout (*Oncorynchus mykiss*). J. Appl. Sci. Res..

[50] FAO The Nutrition and Feed of Farmed Fish and Shrimps. https://www.fao.org/4/ab470e/ab470e06.htm.

[51] Borgese L., Bilo F., Dalipi R., Bontempi E., Depero L.E. (2015). Total Reflection X-Ray Fluorescence as a Tool for Food Screening. Spectrochim. Acta Part B At. Spectrosc..

[52] Oteri M., Rosa A.R.D., Presti V.L., Giarratana F., Toscano G., Chiofalo B. (2021). Black Soldier Fly Larvae Meal as Alternative to Fish Meal for Aquaculture Feed. Sustainability.

[53] Suryati T., Julaeha E., Farabi K., Ambarsari H., Hidayat A.T. (2023). Lauric Acid from the Black Soldier Fly (*Hermetia lllucens*) and Its Potential Applications. Sustainability.

[54] Fabrikov D., Barroso F.G., Sánchez-Muros M.J., Hidalgo M.C., Cardenete G., Tomás-Almenar C., Melenchón F., Guil-Guerrero J.L. (2021). Effect of Feeding with Insect Meal Diet on the Fatty Acid Compositions of Sea Bream (*Sparus aurata*), Tench (*Tinca tinca*) and Rainbow Trout (*Oncorhynchus mykiss*) Fillets. Aquaculture.

[55] Bruni L., Randazzo B., Cardinaletti G., Zarantoniello M., Mina F., Secci G., Tulli F., Olivotto I., Parisi G. (2020). Dietary Inclusion of Full-Fat *Hermetia lllucens* Prepupae Meal in Practical Diets for Rainbow Trout (*Oncorhynchus mykiss*): Lipid Metabolism and Fillet Quality Investigations. Aquaculture.

[56] Mancini S., Medina I., Iaconisi V., Gai F., Basto A., Parisi G. (2018). Impact of Black Soldier Fly Larvae Meal on the Chemical and Nutritional Characteristics of Rainbow Trout Fillets. Animal.

[57] Caimi C., Biasato I., Chemello G., Oddon S.B., Lussiana C., Malfatto V.M., Capucchio M.T., Colombino E., Schiavone A., Gai F. (2021). Dietary Inclusion of a Partially Defatted Black Soldier Fly (*Hermetia lllucens*) Larva Meal in Low Fishmeal-Based Diets for Rainbow Trout (*Oncorhynchus mykiss*). J. Anim. Sci. Biotechnol..

[58] Belghit I., Waagbø R., Lock E., Liland N.S. (2019). Insect-based Diets High in Lauric Acid Reduce Liver Lipids in Freshwater Atlantic Salmon. Aquac. Nutr..

[59] Szendrő K., Zotte A.D., Fülöp N., Garamvölgyi J., Tóth K. (2024). Consumer Views on the Healthiness of Meat from Various Animal Species: A Comprehensive Survey Including Fish. Appl. Food Res..

[60] Rodrigues D.P., Ameixa O.M.C.C., Vázquez J.A., Calado R. (2022). Improving the Lipid Profile of Black Soldier Fly (*Hermetia lllucens*) Larvae for Marine Aquafeeds: Current State of Knowledge. Sustainability.

[61] FAO (2010). Fats and Fatty Acids in Human Nutrition. Report of an Expert Consultation. FAO Food Nutr. Pap..

[62] EFSA Panel on Dietetic Products, Nutrition, and Allergies (NDA) (2010). Scientific Opinion on Dietary Reference Values for Fats, Including Saturated Fatty Acids, Polyunsaturated Fatty Acids, Monounsaturated Fatty Acids, Trans Fatty Acids, and Cholesterol. EFSA J..

[63] Chen J., Liu H. (2020). Nutritional Indices for Assessing Fatty Acids: A Mini-Review. Int. J. Mol. Sci..

[64] Zhang X., Ning X., He X., Sun X., Yu X., Cheng Y., Yu R.-Q., Wu Y. (2020). Fatty Acid Composition Analyses of Commercially Important Fish Species from the Pearl River Estuary, China. PLoS ONE.

[65] Aberoumand A., Baesi F. (2023). Evaluation of Fatty Acid-related Nutritional Quality Indices in Processed and Raw (*Lethrinus lentjan*) Fish Fillets. Food Sci. Nutr..

[66] Orban E., Nevigato T., Lena G.D., Casini I., Marzetti A. (2003). Differentiation in the Lipid Quality of Wild and Farmed Seabass (*Dicentrarchus labrax*) and Gilthead Sea Bream (*Sparus aurata*). J. Food Sci..

[67] Borgogno M., Dinnella C., Iaconisi V., Fusi R., Scarpaleggia C., Schiavone A., Monteleone E., Gasco L., Parisi G. (2017). Inclusion of *Hermetia lllucens* Larvae Meal on Rainbow Trout (*Oncorhynchus mykiss*) Feed: Effect on Sensory Profile According to Static and Dynamic Evaluations. J. Sci. Food Agric..

[68] Lock E.R., Arsiwalla T., Waagbø R. (2016). Insect Larvae Meal as an Alternative Source of Nutrients in the Diet of Atlantic Salmon (Salmo Salar) Postsmolt. Aquac. Nutr..

[69] Sealey W.M., Gaylord T.G., Barrows F.T., Tomberlin J.K., McGuire M.A., Ross C., St-Hilaire S. (2011). Sensory Analysis of Rainbow Trout, Oncorhynchus Mykiss, Fed Enriched Black Soldier Fly Prepupae, *Hermetia lllucens*. J. World Aquac. Soc..

