# Supplementary material for: InsectFish—The Use of Insect Meal in the Fish Sector in Creating Farm-to-Fork Value: Chemical and Quality Characteristics of Sparus aurata Fillets Fed Hermetia illucens Larvae-Based Feed
Source: Foods. 2025 Sep 5;14(17):3107. doi: 10.3390/foods14173107 (PMC12428275; doi:10.3390/foods14173107)
Supplement: Supplementary file 1 [file foods-14-03107-s001.zip › foods-3778656-supplementary.pdf]

Duo-Trio test – raw fish	
N° Assessor:	Date:
You have been given a set of three samples: one reference sample (RIF) and two other samples, one identical to the reference sample (RIF) and one that is different.<br>Please, proceed with the sensory evaluation (visual and olfactory) of the three samples, following the presentation order from left to right, where the first sample is the reference sample (RIF).<br>Circle the identification number of the sample that you believe is different from the reference sample (RIF). Then, please, provide a brief explanation of your selection (differences in appearance and/or odor) and indicate the intensity of the difference (very slight, slight, moderate, noticeable, or absolutely noticeable).<br>This indication must be provided even if, according to your perception, the three samples do not differ.	
Circle the identification number of the sample that you believe is different from the reference sample (RIF)	
Please, briefly explain your selection:	

**Duo-Trio test – cooked fish**	
N° Assessor:	Date:
You have been given a set of three samples: one reference sample and two other samples, one identical to the reference sample (RIF) and one that is different.<br>Please, proceed with tasting the three samples in the order from left to right, where the first sample is the reference sample (RIF). Before tasting each next sample, ensure that you have finished the previous one.<br>Circle the identification number of the sample that you believe is different from the reference sample (RIF). Then, please, provide a brief explanation of your selection (differences in appearance and/or odor and/or taste) and indicate the intensity of the difference (very slight, slight, moderate, noticeable, or absolutely noticeable).<br>This indication must be provided even if, according to your perception, the three samples do not differ.<br>We ask you to drink water and eat half a cracker before starting and also between samples.	
Circle the identification number of the sample that you believe is different from the reference sample (RIF)	
Please, briefly explain your selection:	
